# A Multicenter Retrospective Cohort Study Evaluating the Clinical Outcomes of Patients with Coagulopathy Undergoing Transcatheter Arterial Embolization (TAE) for Acute Non-Neurovascular Bleeding

**DOI:** 10.3390/medicina59071333

**Published:** 2023-07-19

**Authors:** Roberto Minici, Federico Fontana, Massimo Venturini, Giuseppe Guzzardi, Filippo Piacentino, Marco Spinetta, Bernardo Bertucci, Raffaele Serra, Davide Costa, Nicola Ielapi, Andrea Coppola, Pasquale Guerriero, Biagio Apollonio, Rita Santoro, MGJR Research Team, Luca Brunese, Domenico Laganà

**Affiliations:** 1 Radiology Unit, Dulbecco University Hospital, 88100 Catanzaro, Italy; miniciroberto@gmail.com (R.M.); bernardo.bertucci118@gmail.com (B.B.); 2 Diagnostic and Interventional Radiology Unit, ASST Settelaghi, Insubria University, 21100 Varese, Italy; federico.fontana@uninsubria.it (F.F.); massimo.venturini@uninsubria.it (M.V.); filippo.piacentino@asst-settelaghi.it (F.P.); andrea.coppola@asst-settelaghi.it (A.C.); 3 School of Medicine and Surgery, Insubria University, 21100 Varese, Italy; 4 Radiology Unit, Maggiore della Carità University Hospital, 28100 Novara, Italy; giuguzzardi@gmail.com (G.G.); marcospinetta90@gmail.com (M.S.); 5 Vascular Surgery Unit, Department of Medical and Surgical Sciences, Magna Graecia University of Catanzaro, Dulbecco University Hospital, 88100 Catanzaro, Italy; rserra@unicz.it; 6 Department of Law, Economics and Sociology, Magna Graecia University of Catanzaro, 88100 Catanzaro, Italy; davide.costa@unicz.it; 7 Department of Public Health and Infectious Disease, Sapienza University of Rome, 00185 Rome, Italy; nicola.ielapi@uniroma1.it; 8 Radiology Unit, Santobono-Pausilipon Hospital, 80129 Naples, Italy; pasqualeguerriero@gmail.com; 9 Department of Medicine and Health Sciences, University of Molise, 86100 Campobasso, Italy; luca.brunese@unimol.it; 10 Radiology Unit, San Timoteo Hospital, 86039 Termoli, Italy; bapollonio@sirm.org; 11 Haemophilia and Thrombosis Center, Dulbecco University Hospital, 88100 Catanzaro, Italy; ritacarlottasantoro@gmail.com; 12 Magna Graecia Junior Radiologists Research Team, 88100 Catanzaro, Italy; radiologyumg@gmail.com; 13 Scientific Committee of the Italian National Institute of Health (Istituto Superiore di Sanità, ISS), 00161 Rome, Italy; 14 Department of Experimental and Clinical Medicine, Magna Graecia University of Catanzaro, 88100 Catanzaro, Italy

**Keywords:** coagulopathy, bleeding, TAE, embolization, hemorrhage, embolic agents, endovascular

## Abstract

*Background and Objectives*: Transcatheter arterial embolization (TAE) is the mainstay of treatment for acute major hemorrhage, even in patients with coagulopathy and spontaneous bleeding. Coagulopathy is associated with worsening bleeding severity and higher mortality and clinical failure rates. Furthermore, some unanswered questions remain, such as the definition of coagulopathy, the indication for TAE or conservative treatment, and the choice of embolic agent. This study aims to assess the efficacy and safety of TAE for spontaneous non-neurovascular acute bleeding in patients with coagulopathy. *Materials and Methods*: This study is a multicenter analysis of retrospectively collected data of consecutive patients with coagulopathy who had undergone, from January 2018 to May 2023, transcatheter arterial embolization for the management of spontaneous hemorrhages. *Results*: During the study interval (January 2018–May 2023), 120 patients with coagulopathy underwent TAE for spontaneous non-neurovascular acute bleeding. The abdominal wall was the most common bleeding site (72.5%). The most commonly used embolic agent was polyvinyl alcohol (PVA) particles or microspheres (25.0%), whereas coils and gelatin sponge together accounted for 32.5% of the embolic agents used. Technical success was achieved in all cases, with a 92.5% clinical success rate related to 9 cases of rebleeding. Complications were recorded in 12 (10%) patients. Clinical success was significantly better in the group of patients who underwent correction of the coagulopathy within 24 h of TAE. *Conclusions*: Transcatheter arterial embolization (TAE) is effective and safe for the management of acute non-neurovascular bleeding in patients with coagulopathy. Correction of coagulopathy should not delay TAE and vice versa, as better clinical outcomes were noted in the subgroup of patients undergoing correction of coagulopathy within 24 h of TAE.

## 1. Introduction

Transcatheter arterial embolization (TAE) is the mainstay of treatment for acute major hemorrhage [1,2,3]. A multitude of conditions, including trauma, tumors, iatrogenic injuries, and coagulation disorders, can cause major acute bleeding and may benefit from TAE [4,5,6,7,8,9,10,11,12,13,14,15,16,17,18,19,20,21,22,23,24,25,26,27,28,29,30].

Despite TAE in patients with coagulation disorders being a well-known area of endovascular therapy, the definition of coagulopathy differs among authors [4,19,31,32]. The Cardiovascular and Interventional Radiological Society of Europe (CIRSE) and the Society of Interventional Radiology (SIR) issued some documents on peri-operative anticoagulation management in patients undergoing interventional radiology procedures [33,34,35]. Interestingly, some authors have defined the condition of coagulopathy by choosing some cut-off values that previous guidelines or investigations have used to recommend the correction of coagulation function in order to safely perform an endovascular procedure [19,32]. However, the coagulopathy condition in patients undergoing TAE is a separate clinical setting and goes beyond the safety issues related to the endovascular treatment in itself and vascular access site management, as it results in a number of well-known consequences: (1) worsening of the severity of bleeding [36,37], (2) predicting mortality [37] and rebleeding [24,38], (3) higher risk of clinical failure after TAE [39], and (4) high clinical failure rates with embolic agents that act by clot formation, such as gelatin sponge and coils [4,32,40,41,42,43,44].

Moreover, the indication for TAE in patients with coagulopathy is another controversial point. Previous reports demonstrated that conservative management could also be effective in patients with coagulopathy and spontaneous hemorrhages [45,46]. Reversal or withdrawal of anticoagulation therapy, correction of acid–base imbalance, volume resuscitation, vasoactive drugs, transfusions, and correction of coagulopathy with fresh frozen plasma and prothrombin complex concentrate are the main conservative interventions described in previous reports [10,32,39,45,46,47]. However, hemodynamic instability, detection of active bleeding on CT-angiography (CTA), and hematoma volume are the main predictors of unsuccessful conservative management; furthermore, failure of conservative management should be previously recognized [10,45,48,49]. 

Determining the cause of coagulopathy is crucial, given the possibility of initial conservative management. Coagulopathies recognize congenital or acquired causes. Among the acquired causes, some conditions, such as COVID-19-related coagulopathy, thromboprophylaxis with anticoagulants, and trauma-induced coagulopathy, are of particular interest, as they are frequent causes of acute major bleeding requiring TAE [19,50,51,52]. 

Unfortunately, the existing evidence on the safety and efficacy of TAE in patients with coagulopathy is inconsistent; often limited to small populations, retrospective-based case series [19], or subgroup analyses; and performed in a single-center fashion [31,32,39,44,53,54]. Our study aims to assess the efficacy and safety of TAE for spontaneous non-neurovascular acute bleeds in patients with coagulopathy via a multicenter, retrospective, large cohort evaluation. 

## 2. Materials and Methods

### 2.1. Study Design

**Figure 1:**
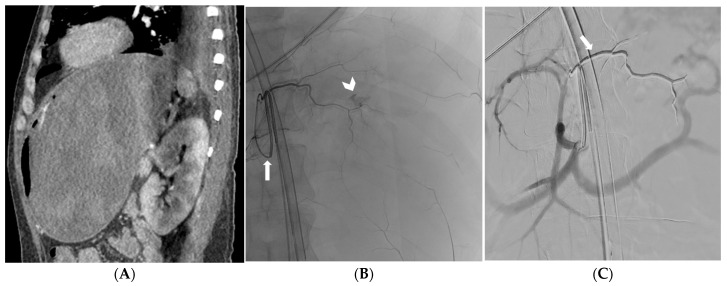
CT angiography reformatted image in the oblique sagittal plane depicting recurrent massive gastric bleeding after endoscopic treatment (**A**). Selective catheterization of the left gastric artery (arrow) with active bleeding confirmed at fluoroscopy (arrowhead) (**B**). Digital subtraction angiography showing effective embolization and Onyx 18 cast (arrow) distributed along the left gastric artery (**C**).

**Figure 2:**
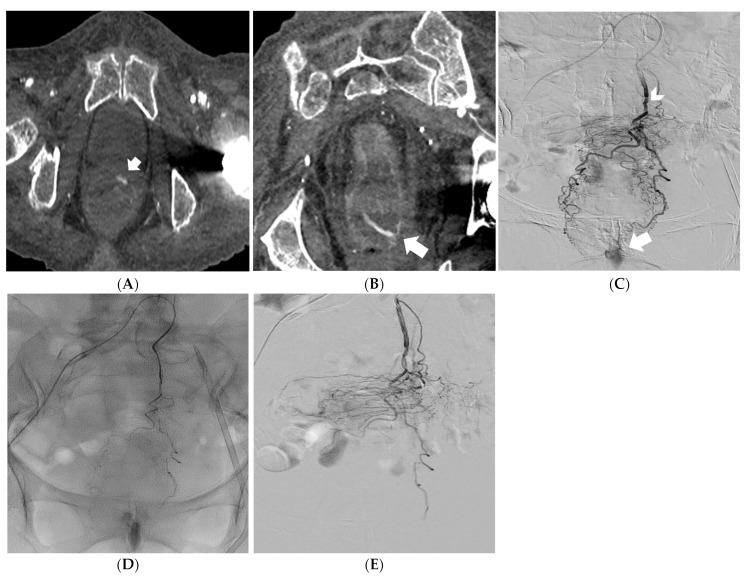
Reformatted CT angiography images in the oblique axial and coronal planes showing a massive rectal hemorrhage (arrows) (**A**,**B**). Digital subtraction angiography identifying the superior rectal artery as the feeding vessel (arrowhead); active bleeding is also noted (arrow) (**C**). Embolization of the superior rectal artery with PVA particles mixed with iodinated contrast media under fluoroscopy control (**D**). Digital subtraction angiography confirming successful embolization of the feeding vessel (patency of the middle and inferior rectal arteries, fed by the internal iliac arteries, prevented significant ischemic injury of the rectal wall) (**E**).

**Figure 3:**
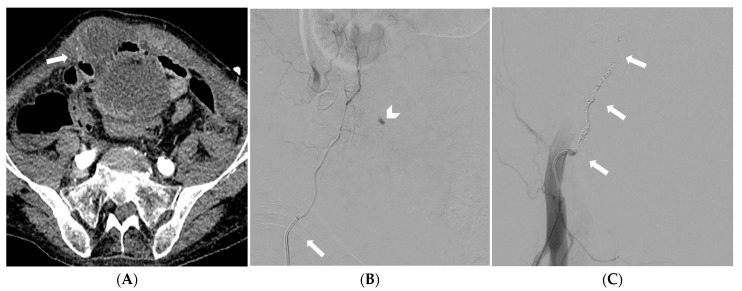
CT angiography depicting a spontaneous rectus sheath hematoma (arrow) (**A**). Selective catheterization of the right inferior epigastric artery (arrow) with a pseudoaneurysm confirmed at digital subtraction angiography (arrowhead) (**B**). Digital subtraction angiography showing effective embolization with coil (arrows) embolization starting distal to a small pseudoaneurysm-feeding vessel, thus preventing “backdoor” bleeding (**C**).

**Figure 4:**
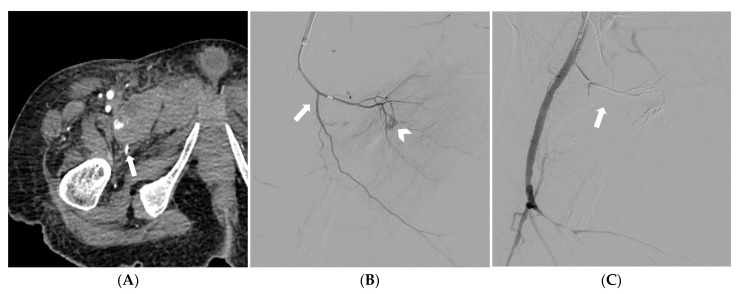
CT angiography depicting a spontaneous pectineus muscle hematoma with active bleeding (arrow) (**A**). Selective catheterization of the right profunda femoris artery and superselective catheterization of the medial circumflex femoral artery (arrow); at digital subtraction angiography active bleeding is noted (arrowhead) (**B**). Digital subtraction angiography demonstrating successful embolization with a gelatin sponge (arrow); preserved patency of the profunda femoris artery and its other branches is also noted (**C**).

This study is a multicenter (Dulbecco University Hospital, Catanzaro, Italy; Circolo Hospital, Varese, Italy; Maggiore della Carità University Hospital, Novara, Italy; Mater Domini University Hospital, Catanzaro, Italy; Pugliese-Ciaccio Hospital, Catanzaro, Italy; Cardarelli Hospital, Campobasso, Italy; San Timoteo Hospital, Termoli, Italy) analysis of retrospectively collected data of consecutive patients with coagulopathy who underwent, from January 2018 to May 2023, transcatheter arterial embolization for the management of acute bleeding (Figure 1, Figure 2, Figure 3 and Figure 4). Inclusion criteria were (I) TAE performed due to spontaneous non-neurovascular acute bleeding according to the indications provided by the Society of Interventional Radiology (SIR) guidelines for percutaneous transcatheter embolization [3], (II) coagulopathy at the time of embolization, (III) age of at least 18 years, and (IV) evaluation by a multidisciplinary team of surgeons, interventional radiologists and anesthesiologists. The exclusion criteria were (I) pregnant or breastfeeding women; (II) platelet count < 20,000/μL and refusal of transfusion, according to SIR guidelines for low bleeding risk procedures that require arterial access [34]; (III) international normalized ratio (INR) greater than 1.8 for femoral access or greater than 2.2 for radial access, according to SIR guidelines for low bleeding risk procedures that require arterial access [34]; (IV) hypersensitivity to available embolic agents; and (V) bleeding sustained by the internal carotid artery or its branches.

In order to constitute a control group, we retrospectively examined consecutive patients with normal coagulation function who underwent TAE to manage acute non-neurovascular bleeding, starting from January 2018 until equal sample size of the group of patients with coagulopathy was constituted. Ethics committee approval was not required due to the retrospective nature of the study. The study was conducted in accordance with the Helsinki Declaration. All patients signed a written informed consent before receiving the endovascular treatment. 

### 2.2. Treatment 

All patients underwent pre-treatment evaluation with CT-angiography (CTA), except in special cases, based on international guidelines or expert opinions (e.g., upper gastrointestinal bleeding refractory to endoscopic treatment [55]). The endovascular procedure was performed in dedicated angiographic suites by an experienced interventional radiologist (at least 5 years of experience). A high-level infection protection protocol was adopted before, during, and after the endovascular procedure for COVID-19 patients, similar to what was suggested by Ierardi et al. [56]. Diagnostic angiography always preceded the super-selective catheterization of the bleeding/pseudoaneurysm-feeding arteries. The choice of embolic agent depended on operator preference. The embolic agent was prepared and injected under fluoroscopic guidance according to the instructions for use. The microcatheter was never reused or washed after the use of a non-adhesive liquid embolic agent (NALEA) or N-butyl cyanoacrylate (NBCA). In the absence of angiographic signs of bleeding, a blind embolization was performed [48]. Assessment of technical success and non-target embolization were performed by postembolization angiography, taking into account the possible collateral circulation based on the anatomical site of the bleeding. When appropriate, the anesthesiologist performed sedation during the embolization to improve patient comfort and provided analgesic therapy after the procedure. All patients underwent clinical evaluation and follow-up imaging before the hospital discharge and 1 month after TAE.

### 2.3. Outcomes and Definitions

The primary efficacy endpoint was the rate of technical success. The clinical success rate was selected as a secondary efficacy endpoint. The primary safety endpoint was the rate of complications. The non-target embolization rate and the rate of major complications graded according to the 2003 SIR classification [57] were selected as secondary safety endpoints. 

Unless otherwise specified, reporting standards of the Society for Interventional Radiology for percutaneous transcatheter embolization were used (e.g., technical success was defined by the exclusion defined by angiographically appreciable bleeding stopping immediately after embolization; the resolution of the signs of bleeding, which made TAE necessary, defined the clinical success) [58]. The coagulopathy was defined as in Loffroy et al. [4]: INR greater than 1.5, partial thromboplastin time longer than 45 s, or platelet count less than 80,000/mm^3^. Ct-to-groin time, procedure time, and CT-to-embolization time were calculated by taking into account the times indicated in the CT report and the surgical operative log. Complications linked to TAE were graded according to the 2017 SIR classification [59], the 2003 SIR classification [57], and the CIRSE classification [60].

### 2.4. Statistical Analysis

Data were maintained in an Excel spreadsheet (Microsoft Inc., Redmond, WA, USA) and the statistical analyses were performed on an intention-to-treat basis, using SPSS software (SPSS, version 22 for Windows; SPSS Inc., Chicago, IL, USA) and R/R Studio software. The analyses were based on the modified intention-to-treat population, defined as all randomized patients who received at least one embolization treatment [61,62]. The Kolmogorov–Smirnov test and the Shapiro–Wilk test were used to verify the normality assumption of data. Categorical data are presented as frequencies (percentage value). Continuous normally distributed data are presented as means ± standard deviations. Continuous not normally distributed data are presented as medians (first–third quartile) [63,64,65]. The unpaired Student’ *t*-test was used to assess statistical differences for continuous normally distributed data [66], whereas categorical and continuous not normally distributed data were assessed using the Chi-squared/Fisher’s exact tests and the Mann–Whitney test, respectively [67,68]. A *p*-value of <0.05 was considered statistically significant for the aforementioned tests.

## 3. Results

During the study interval (January 2018–May 2023), 120 patients with coagulopathy underwent transcatheter arterial embolization for spontaneous non-neurovascular acute bleeding. An INR > 1.5 was observed in 72 (60.0%) patients, an aPTT > 45 s in 72 (60.0%) patients, and a PLT value below 80,000/mm^3^ in 66 (55.0%) patients. CT-angiography was performed in 117 (97.5%) cases and bleeding was detected in 111 (92.5%) patients. The mean hematoma volume measured on CT was 238.6 (±263.3) mL. In total, 99 (82.5%) patients were on antiplatelet or anticoagulant therapy. Details are given in Table 1.

120 transcatheter arterial embolizations were performed. In three cases no bleeding was detected on X-ray angiography (XA); thus blind embolizations were performed (i.e., embolization of lumbar arteries guided by CT findings). The abdominal wall was the most common bleeding site (72.5%). The most commonly used embolic agent was polyvinyl alcohol (PVA) particles or microspheres (25.0%), whereas coils and gelatin sponge together account for 32.5% of the embolic agents used. In 27 (22.5%) cases Onyx or Squid was used. The mean volume of iodinated contrast media used during embolizations was 38.1 (±14) mL. The mean volume-of-contrast-to-creatinine-clearance ratio was 0.73 (±0.7). The most common vascular access site was the common femoral artery (70%). The mean CT-to-groin time, the mean procedure time, the mean CT-to-embolization time, and the mean fluoroscopy time were 52.8 (±58.7) min, 30.7 (±10.5) min, 83.8 (±59.2) min, and 8.3 (±3.4) min, respectively. Radiation exposure expressed by cumulative air kerma and total dose area product was 159.7 (±62) mGy and 25.5 (±9.7) Gy/cm^2^, respectively.

Procedure data are detailed in Table 2.

Technical success was achieved in all cases, with a 92.5% clinical success rate related to 9 cases of rebleeding. No cases of non-target embolization were observed. Complications were recorded in 12 (10%) patients. Vascular access site complications (VASCs) were recorded in six (5%) cases, related to three pseudoaneurysms and three groin hematomas. Furthermore, three liver abscesses and three psoas abscesses were noted. According to the 2017 SIR classification for complications [59], all complications observed were grade 2 events. The 30 day bleeding-related mortality was 5%, related to 6 cases of hypovolemic shock and multiple organ dysfunction syndrome (MODS); another 6 (5%) patients died within the deadline of the 30 day follow-up from causes not related to the bleeding.

Details are given in Table 3.

No statistically significant differences were observed between Group 1 (patients who did not undergo correction of the coagulopathy within 24 h of the TAE) and Group 2 (patients who underwent correction of the coagulopathy within 24 h of the TAE) in terms of preoperative INR, platelet count, procedure time, technical success, and complications. Statistically significant differences were observed between Group 1 and Group 2 in terms of preoperative hemoglobin, anticoagulant therapy, hematoma volume, CT-to-groin time, fluoroscopy time, clinical success, rebleeding rate, and 30 day bleeding-related mortality.

A comparison of data between Group 1 and Group 2 is reported in Table 4.

No statistically significant differences were observed between patients with coagulopathy undergoing TAE for acute non-neurovascular bleeding and the control group consisting of patients with normal coagulation function in terms of preoperative hemoglobin (*p* = 0.397), procedure time (*p* = 0.141), technical success (*p* = 0.247), clinical success (*p* = 0.595), rebleeding rate (*p* = 0.811), complications (*p* = 0.649), or 30 day bleeding-related mortality (*p* = 0.758). Preoperative INR (*p* < 0.001), platelet count (*p* < 0.001), and age (*p* = 0.009) were not comparable between the abovementioned groups. Details are reported in Table 5.

## 4. Discussion

In this multicenter retrospective cohort study, acute major non-neurovascular bleeding in patients with coagulopathy was effectively managed by TAE.

Technical success was achieved in all cases, with a clinical success rate of 92.5%; 9 cases of rebleeding were all successfully retreated with TAE. These results are in keeping with previous investigations evaluating TAE in acute bleeding of patients with coagulopathy [19,31,32,39,44,53,54]. Spiliopoulos et al. reported a technical success rate of 100% in their case series of 17 patients with coagulopathy who underwent TAE for acute bleeding [19]. Interestingly, they successfully used gelatin sponge, coils, or a combination of both in eight (47.1%) cases. The only rebleeding event was observed in a patient treated with gelatin sponge alone, thus recording a rebleeding rate of 5.9% [19]. In a recent case series of 35 cirrhotic patients by Patidar et al., 91.4% of patients had coagulopathy, and technical and clinical success was achieved in 100% and 82.8% of patients, respectively [31]. Yonemitsu et al. achieved primary hemostasis in 50 of the 63 bleeding arteries (80%) in patients with a coagulopathic condition. Interestingly, the primary hemostatic rates in the gelatin sponge, coils, and NBCA groups were 67% (18 of 27), 80% (16 of 20), and 100% (16 of 16), respectively. Secondary hemostasis was achieved in 60 of the 63 hemorrhagic arteries (95%) [32]. According to Jae et al., TAE with NBCA was associated with a high clinical success rate (83%) in patients with coagulopathy [69]. Less recent reports have shown lower technical success rates, such as 43% reported by Encarnacion et al. [70] and 46% reported by Schenker et al. [71], demonstrating the gradual improvement of TAE efficacy over time. Hence, we can speculate that, despite the lack of specific guidelines, TAE represents an effective treatment option for acute major hemorrhages in patients with coagulopathy. Finally, our results are in keeping with the control group consisting of patients with normal coagulation function and with outcomes reported in other investigations for acute non-neurovascular bleeding in patients with normal coagulation function [5,6,11,58].

In our study, TAE showed a good safety profile in the treatment of acute non-neurovascular bleeding in patients with coagulopathy. Complication rate and 30 day bleeding-related mortality were 10% and 5%, respectively. Lee et al. demonstrated that coagulopathy is an independent predictor of rebleeding and mortality [38]. Interestingly, Aina et al. pointed out a higher rate of rebleeding in patients with coagulopathy undergoing TAE than in patients with normal coagulation function [72]. Encarnacion et al. and Schenker et al. observed that TAE was 2.9 times and 2.8 times, respectively, more likely to fail in patients with coagulopathy [70,71]. This finding should be put in context with more recent evidence that, with technological advances and better rationale for using embolic agents, has shown a marked improvement in the rebleeding rates. Recently, Spiliopoulos et al. reported no cases of bleeding-related 30 day mortality; in all cases, they used a vascular closure device to achieve vascular access site hemostasis, with no major bleeding-related VASCs or procedure-related complications noted [19]. In their case series on 63 TAEs performed in patients with a coagulopathic condition, Yonemitsu et al. observed a significantly higher rebleeding rate in the gelatin sponge group compared to the coil group (23% vs. 0%; *p* = 0.048), as well as in the gelatin sponge group compared to the NBCA group (23% vs. 0%; *p* = 0.048) [32]. In a recent case series of 35 cirrhotic patients by Patidar et al., 91.4% of patients had coagulopathy; the 30-day mortality rate and bleeding-related mortality rate were 48% and 17%, respectively; and the rebleeding rate was 8.6%. The overall complication rate was 8.5%, with no major procedure-related complications noted. Interestingly, NBCA was used as the embolic agent and only the greater number of RBC units transfused before the embolization procedure was significantly associated with clinical failure [31]. Hence, in our investigation, the safety outcomes, including VASCs, are consistent with other studies in the field of endovascular treatments and TAEs [6,15,25,29,73,74,75,76,77,78,79,80] and with the control group consisting of patients with normal coagulation function.

The heterogeneity of data and some unanswered questions on TAE in patients with coagulopathy should be recognized. Firstly, the definition of coagulopathy may vary according to various authors, as well as the inclusion criteria. Loffroy et al. defined the coagulopathy subgroup as patients having a platelet count of less than 80,000/μL, an international normalized ratio (INR) greater than 1.5, or an activated partial thromboplastin time (aPTT) longer than 45 s or [4]. Yonemitsu et al. defined the condition of coagulopathy as platelet count < 50,000/μL and/or INR < 1.5 because these values required correction by infusion of platelets or fresh frozen plasma according to previous indications [32]. According to the guidelines for periprocedural management of coagulation status and hemostasis risk in percutaneous image-guided interventions issued in 2012 by SIR, procedures requiring an introducer sheath up to 7Fr should be performed after optimization of the coagulation status to the following values: platelet count > 50,000/μL, INR < 1.5, and aPTT < 1.5 x laboratory reference values [81]. These guidelines were cited by other authors to justify their definition of coagulopathy. Spiliopoulos et al. define the coagulopathy subgroup as patients presenting with thrombocytopenia (platelet count < 50,000/μL) and/or INR ≥ 2.0, and/or aPTT > 45 s, and/or a known underlying blood-clotting disorder such as factor VIII, von Willebrand disease, or hepatic cirrhosis with abnormal liver function tests [19]. Similar inclusion criteria were used by Hur et al. [39]. In 2019, SIR issued updated Consensus Guidelines for the Periprocedural Management of Thrombotic and Bleeding Risk in Patients Undergoing Percutaneous Image-Guided Interventions, recommending the correction of INR within the range of ≤2.0–3.0 and a platelet count > 20,000/μL to safely perform low bleeding risk procedures including embolotherapy. Interestingly, the low quality of evidence supporting this recommendation should also be noted [34,35]. International Society on Thrombosis and Haemostasis interim guidance on recognition and management of coagulopathy in COVID-19 defined the condition of coagulopathy in COVID-19 patients as prothrombin time (PT) prolongation above laboratory reference values (may vary depending on the reagent used), D-dimer ≥ 1.5 mg/L, fibrinogen < 2 g/L, or platelet count less than 100,000/μL [50]. Recently, CIRSE issued a standard of practice document on peri-operative anticoagulation management during interventional radiology procedures recommending a platelet count > 20,000/μL and an INR < 2.0 to perform low bleeding risk procedures such as embolization [33]. The above guidelines define cut-off values to safely perform the percutaneous treatment and adequately manage the vascular access site. Instead, the definition of coagulopathy in patients undergoing TAE should also be aimed at directing the choice of embolic agent, since some of them act by clot formation. A previous investigation by Loffroy et al. demonstrated that the use of coils as the sole embolic agent and the presence of coagulopathy are independent predictors of early rebleeding; interestingly, higher cut-off values were used to define the coagulopathy status [4]. Therefore, it is our opinion that the heterogeneity in the coagulopathy definition can lead to errors in comparing published data and that it would be desirable to use a common, broader definition of coagulopathy, such as those proposed by Spiliopoulos et al., Loffroy et al., or the International Society on Thrombosis and Haemostasis [4,19,50]. Future perspectives include the development of a predictive model of the clinical efficacy of various embolic agents based on different values of PLT, aPTT, and INR.

It is noteworthy that 30 day survival after TAE could be predicted by clinical success [82]. Moreover, coagulopathy has been previously reported as a significant negative predictor for recurrent bleeding, in-hospital mortality, and procedure-related complication rates [1,2,24,39,83,84]. Interestingly, a higher risk of rebleeding has been observed in patients with impaired coagulation even after successful TAE [83]. In contrast, other reports have shown similar rates of TAE success and rebleeding between patients with coagulopathy and those without [19]. Additionally, the coronavirus disease 2019 (COVID-19) pandemic has resulted in increased reports on effective conservative management of spontaneous major bleeds in patients with coagulopathy [45,46,85]. Therefore, data on TAE in patients with coagulopathy are controversial, and the management of major acute bleeding in patients with coagulopathy is still debated. Loffroy et al. advocated for TAE for patients with coagulopathy; they argued that endovascular treatment should not be refused and that correction of the coagulopathy should be performed as soon as possible, regardless of TAE [83]. Similarly, if large-volume bleeding causing hemodynamic instability is noted, better clinical outcomes will be expected in case of a shorter time to embolization; waiting for normalization of the coagulation state is not a good option [3]. Hence, TAE should not delay the correction of coagulopathy, and vice versa. Our findings support this speculation, as a reduced rebleeding rate was observed in the subgroup of patients who underwent correction of the coagulopathy within 24 h of TAE. Spiliopoulos et al. speculated that several factors, including local expertise in multidisciplinary bleeding management, correction of coagulopathy, and the timing and technique of TAE, are crucial for improved outcomes. Notably, in their investigation normalization of the coagulation state within 24 h was recorded in 58% of the patients [19]. 

The choice of the best embolic agent is another key point. Previous reports support the hypothesis that embolization with a gelatin sponge or coils only is less effective in patients with coagulopathy, as their mechanism of action is primarily based on clot formation [4,32,40,41,42,43,44]. However, patients with significantly prolonged INR were successfully treated with coil-only embolization [86], and dense coil packing or a combination of dense coiling with gelatin sponge was suggested for patients with coagulopathy undergoing TAE [19]. Coils could guarantee a proximal embolization with reduced risk of migration compared to NBCA, particles, or NALEAs, but in patients with coagulopathy, the dense coil-packing technique is necessary, although it is less user-friendly for less experienced interventionists. Hence, we believe that an imperfect coil embolization technique could jeopardize the outcomes of TAE, especially if there is no strong experience in the dense coil-packing technique. Conversely, particles and liquid embolics guarantee rapid and effective mechanical embolization, not requiring the activation of coagulation [87,88]. PVA particles and microspheres cause mechanical vascular occlusion by depositing in vessels and causing an inflammatory and then a fibrotic reaction in the vessel wall, and they are available in different size ranges, which determine their ability to penetrate even very small vessels [89,90]. Non-adhesive liquid embolic agent (NALEA) and N-butyl cyanoacrylate (NBCA) have been proposed as the most effective embolic agents in patients with coagulopathy, as they act by polymerization (NBCA) and solidification (Onyx), thus embolizing the target vessel independently from the clot formation [44,74,91]. TAE with NBCA has a favorable cost-effectiveness ratio [92], but its use requires a longer learning curve than coils and is probably less user-friendly than non-adhesive liquid embolic agents (NALEAs) due to the higher risk of insidious adverse events such as the gluing of the microcatheter tip [40].

One could argue extensively over the safety management of the vascular access site in patients with coagulopathy. According to SIR guidelines for low bleeding risk procedures that require arterial access, femoral access requires an INR of 1.8 or less, whereas radial access requires an INR of 2.2 or less [34]. Furthermore, the use of vascular closure devices may facilitate hemostasis of the femoral access site, and deferring the removal of the introducer sheath until correction of the coagulopathy may be an option [19]. In our study, the incidence of bleeding-related VASCs was low and comparable to other reports in the literature on TAE and other endovascular treatments in patients with normal coagulation status [6,15,74,76,93,94].

Therefore, our results together with the review of data in the literature allow us to support some speculations regarding patients with coagulopathy and major acute bleeding, which can be summarized as follows: (1) The different definitions of coagulopathy and the variability of the embolic agents used determine a heterogeneity of data in the literature, thus resulting in controversial and difficult-to-compare outcomes; (2) conservative management may be an option but the main predictors of its failure must be considered, including hemodynamic instability, detection of active bleeding on CTA, and hematoma volume; (3) identifying the causes of coagulopathy is crucial given that the correction of coagulopathy improves outcome; (4) TAE is a safe and effective option; (5) TAE should not delay the correction of coagulopathy, and vice versa; (6) the choice of embolic agents not acting by clot formation might promote the achievement of efficacy rates similar to TAE in patients with normal coagulation function; (7) INR guides the choice of vascular access to safely perform TAE; and (8) coagulopathy does not preclude the safe management of vascular access, thanks also to the use of VCDs and the possibility of postponing the removal of the introducer sheath.

Limitations of the study are the retrospectivity of the analysis, the heterogeneity of the indications, the short-term follow-up, and the scarcity of data in the literature, which are necessary to evaluate the congruence and the consistency of the data presented. Besides, as this is a retrospective study, the sample size calculation could not be performed, and thus, the statistical power of the study may not be optimal.

## 5. Conclusions

The results of the current investigation demonstrate that transcatheter arterial embolization (TAE) is an effective, safe, and potentially life-saving option for the management of acute non-neurovascular bleeding in patients with coagulopathy. Correction of coagulopathy should not delay TAE, and vice versa, as better clinical outcomes were noted in the subgroup of patients undergoing correction of coagulopathy within 24 h of TAE.

Further studies are warranted to better understand the impact on outcomes of some factors, such as the choice of the best embolic agent, the indications to TAE, and the time to angiography in patients with coagulopathy.

## Figures and Tables

**Table 1:** Population data.

Variables	All Patients (*n* = 120)
Age (years)	63.6 (±15.9)
Sex (M/F)	78 (65.0%)/42 (35%)
BMI	26.4 (±3.7)
eGFR (mL/min)	68.7 (±24.1)
CKD Stage	2 (1–3)
INR	1.6 (±0.3)
aPTT (s)	46.2 (±5.7)
PT (s)	15.8 (±2.6)
Platelet count (No. ×10^3^/μL)	206.7 (±163.6)
Coagulopathy - INR > 1.5 - aPTT > 45 s - PLT < 80,000/mm^3^	72 (60.0%) 72 (60.0%) 66 (55.0%)
Hemoglobin (g/dL)	7.8 (±0.8)
CT-angiography execution	117 (97.5%)
Bleeding on CT-angiography	111 (92.5%)
Hematoma volume (mL)	238.6 (±263.3)
Antiplatelet therapy - Single - Dual	18 (15.0%) 12 (10.0%) 5 (5%)
Anticoagulant therapy	81 (67.5%)
Antiplatelet AND anticoagulant therapy	0 (0%)
Antiplatelet OR nticoagulant therapy	99 (82.5%)

**Table 2:** Procedure data.

Variables	All Patients (*n* = 120)
Bleeding on XA	117 (97.5%)
Blind embolization	3 (2.5%)
Site of bleeding - Pelvic - Splanchnic organs - Abdominal wall - Thorax - Limbs	3 (2.5%) 12 (10.0%) 87 (72.5%) 12 (10.0%) 6 (5.0%)
Number of embolized vessels	1.1 (±0.4)
Embolic agent - Gelatin sponge - Coils - PVA particles or microspheres - NBCA - Onyx or Squid	12 (10.0%) 27 (22.5%) 30 (25.0%) 24 (20.0%) 27 (22.5%)
Intraoperative contrast medium (mL)	38.1 (±14)
Volume-of-contrast-to-creatinine-clearance ratio	0.73 (±0.7)
Vascular access site - Femoral - Radial - Brachial	84 (70.0%) 30 (25.0%) 6 (5%)
Sheath diameter, 4F/5F/6F/≥7F	15 (12.5%)/102 (85%)/3 (2.5%)/0 (0%)
CT-to-groin time (min)	52.8 (±58.7)
Procedure time (min)	30.7 (±10.5)
CT-to-embolization time (min)	83.8 (±59.2)
Fluoroscopy time (min)	8.3 (±3.4)
Cumulative air kerma (mGy)	159.7 (±62)
Dose area product (DAP) (Gy/cm^2^)	25.5 (±9.7)

**Table 3:** Outcome data.

Variables	All Patients (*n* = 120)
Technical success	120 (100%)
Clinical success	111 (92.5%)
Coagulopathy correction within 24 h of TAE	57 (47.5%)
Vascular access site hemostasis - Manual compression - Vascular closure device	63 (52.5%) 57 (47.5%)
Units of packed red blood cells transfused per patient	1.4 (±1.2)
Rebleeding	9 (7.5%)
Non-target embolization	0 (0%)
Complications	12 (10%)
Vascular access-site complications (VASCs)	6 (5%)
Complications, according to SIR classifications - None - Minor (grades 1 and 2) - Major (grades 3–5)	108 (90%) 12 (10%) 0 (0%)
Complications, according to CIRSE classification - None - Grade 3	108 (90%) 12 (10%)
Treatment required for complications - None - Medical - Interventional - Surgical	108 (90%) 6 (5%) 6 (5%) 0 (0%)
30 day bleeding-related mortality	6 (5%)

**Table 4:** Comparison of data between Group 1 (patients who did not undergo correction of the coagulopathy within 24 h of the TAE) and Group 2 (patients who underwent correction of the coagulopathy within 24 h of the TAE).

Variables	Group 1 (*n*° = 63)	Group 2 (*n*° = 57)	*p*-Value
Preoperative INR	1.59 (±0.3)	1.52 (±0.3)	0.242
Platelet count (No. ×10^3^/μL)	202.4 (±166)	211.3 (±162.3)	0.9263
Preoperative hemoglobin (g/dL)	7.6 (±0.5)	7.9 (±0.9)	0.0419
Anticoagulant therapy	27 (42.9%)	54 (94.7%)	<0.0001
Hematoma volume (mL)	183.4 (±253.7)	303.5 (±261.9)	<0.0001
CT-to-groin time (min)	41.6 (±46.4)	64.6 (±67.8)	0.0107
Procedure time (min)	29.1 (±10.2)	32.4 (±10.6)	0.0523
Fluoroscopy time (min)	7.7 (±2.8)	9 (±3.8)	0.0135
Technical success	63 (100%)	57 (100%)	1
Clinical success	54 (85.7%)	57 (100%)	0.0031
Rebleeding rate	9 (14.3%)	0 (0%)	0.0031
Complications	9 (14.3%)	3 (5.3%)	0.1317
30 day bleeding-related mortality	6 (9.5%)	0 (0%)	0.0285

**Table 5:** Comparison of data between patients with coagulopathy and a control group consisting of patients with normal coagulation function.

Variables	Coagulopathy (*n*° = 120)	Normal Coagulation Function (*n*° = 120)	*p*-Value
Age (years)	63.6 (±15.9)	55.9 (±20.3)	0.009
Preoperative INR (s)	1.6 (±0.3)	1.3 (±0.3)	<0.001
Platelet count (No. ×10^3^/μL)	206.7 (±163.6)	357.4 (±111)	<0.001
Preoperative hemoglobin (g/dL)	7.8 (±0.8)	7.7 (±0.8)	0.397
Procedure time (min)	30.7 (±10.5)	28.4 (±9)	0.141
Technical success	120 (100%)	117 (97.5%)	0.247
Clinical success	111 (92.5%)	114 (95%)	0.595
Rebleeding rate	9 (7.5%)	10 (8.3%)	0.811
Complications	12 (10%)	9 (7.5%)	0.649
30 day bleeding-related mortality	6 (5%)	5 (4.2%)	0.758

## Data Availability

The data presented in this study are available on request from the corresponding author. The data are not publicly available due to privacy issues.

## Abbreviations

APTT: activated partial thromboplastin time; CKD: chronic kidney disease; CIRSE: Cardiovascular and Interventional Radiological Society of Europe; COVID-19: coronavirus disease 2019; CTA: CT-angiography; DAP: dose–area product; eGFR: estimated glomerular filtration rate; EVOH: ethylene-vinyl alcohol; INR: international normalized ratio; MODS: multiple organ dysfunction syndrome; NALEA: non-adhesive liquid embolic agent; NBCA: N-butyl cyanoacrylate; PT: prothrombin time; PVA: polyvinyl alcohol; SIR: Society of Interventional Radiology; TAE: transcatheter arterial embolization; VASCs: vascular access site complications; XA: X-ray angiography.
